# Comments on ‘Association of FcϵRIβ polymorphisms with risk of asthma and allergic rhinitis: evidence based on 29 case–control studies’

**DOI:** 10.1042/BSR20193424

**Published:** 2020-07-21

**Authors:** Haijun Yang, Lan Zheng, Yanmei Zhang, Min Yang, Sha Wei

**Affiliations:** 1 Department of Preventive Medicine, College of Basic Medical Sciences, Hubei University of Chinese Medicine, Hongshan District, Wuhan 430065, P.R. China; 2 Department of Internal Medicine, the First School of Clinical Medicine, Hubei University of Chinese Medicine, Wuchang District, Wuhan 430060, P.R. China; 3 Section of Respiratory Medicine, Department of Internal Medicine, Hubei Provincial Hospital of Traditional Chinese Medicine, Wuchang District, Wuhan 430061, P.R. China

**Keywords:** allergic rhinitis, asthma, FcERIB, meta analysis

## Abstract

Guo *et al*. (*Bioscience Reports* (2018) **38**, BSR20180177) published a meta-analysis concerning the association between five single nucleotide polymorphisms (SNPs) in the high-affinity IgE receptor β chain (*FcεRIβ*) gene, namely E237G, -109 C/T, RsaI_in2, RsaI_ex7, and I181L, and risk of asthma and allergic rhinitis based on available 29 case–control studies. Summary odds ratios (ORs) and 95% confidence intervals (CIs) were used to assess the strength of association of SNPs in *FcεRIβ* gene with allergic diseases risk. They found that *FcεRIβ* E237G (237G vs. 237E: OR = 1.28, 95% CI = 1.06–1.53) and −109 C/T (TT vs. CT+CC: OR = 1.58, 95% CI = 1.26–1.98) were risk factors for allergic diseases. Guo *et al*.’s findings are interesting, but we found that several issues should be clarified after carefully reading the paper. Here, we intended to comment on these data clarifications.

Dear editor,

We researched the relevant studies about the association between the high-affinity IgE receptor β chain (*FcεRIβ*) polymorphisms and allergic diseases risk in Medline, Embase, Web of Science, Chinese National Knowledge Infrastructure, and Wanfang databases. No limit of start year and month was set, and the updated time was August 2019. The terms, search strategies, and inclusion/exclusion criteria were the same as reported by Guo et al. [1]. Comparing our retrieved studies with the ones in Table 1 of Guo *et al*.’s paper [1], it seems that some errors or mistakes should be corrected.

**Table 1:** Main characteristics of eligible studies

Author	Year	Country	Ethnicity	Atopy	Sample size (*n*)		Genotype frequency (*n*)						HWE (*P*)
					Case	Control	Case			Control			
							EE	EG	GG	EE	EG	GG	
*FcεRIβ* gene E237G polymorphism													
Shirakawa	1996	Japan	Asian	asthma	300	100	256	44	0	94	6	0	1.000
Green	1998	South Africa	African	asthma	41	42	27	12	2	25	17	0	0.172
Green	1998	South Africa	Caucasian	asthma	46	51	35	11	0	47	4	0	1.000
Rohrbach	1998	Switzerland	Caucasian	asthma	224	159	207	17	0	151	8	0	1.000
Ishizawa	1999	Japan	Asian	asthma	90	102	70	19	1	81	21	0	0.593
Chen	2000	China	Asian	asthma	101	47	59	39	3	30	16	1	1.000
Soriano	2000	Spain	Caucasian	asthma	145	47	134	11	0	43	4	0	1.000
Takabayashi	2000	Japan	Asian	asthma	100	100	69	27	4	65	33	2	1.000
Nagata	2001	Japan	Asian	rhinitis	233	100	150	76	7	77	18	5	0.021
Zeng	2001	China	Asian	asthma	69	28	61	5	3	27	1	0	1.000
Cui	2003	China	Asian	asthma	216	198	125	80	11	148	46	4	0.766
Korzycka	2004	Poland	Caucasian	asthma rhinitis	98	87	92	6	0	83	4	0	1.000
Rigoli	2004	Italy	Caucasian	asthma rhinitis	100	103	79	16	5	102	1	0	1.000
Sharma	2004	India	Asian	asthma	329	266	300	29	0	250	16	0	1.000
Zhang (Chinese)	2004	Singapore	Asian	asthma	141	157	81	57	3	108	42	7	1.194
Zhang (Indian)	2004	Singapore	Asian	asthma	82	98	71	10	1	80	18	0	1.000
Zhang (Malay)	2004	Singapore	Asian	asthma	68	100	49	19	0	77	23	0	0.353
Zhao	2004	China	Asian	asthma	151	105	126	23	2	92	13	0	1.000
Kim	2006	Korea	Asian	asthma	307	264	235	64	8	177	81	6	0.353
Li	2006	China	Asian	asthma	50	40	43	7	0	40	0	0	1.000
Liu	2006	China	Asian	asthma	60	50	45	14	1	39	10	1	0.527
Kim	2009	Korea	Asian	asthma	347	303	244	99	4	217	81	5	0.409
Wang	2009	China	Asian	asthma	446	506	309	121	16	314	165	27	0.386
Undarmaa	2010	Japan	Asian	asthma	367	630	256	102	9	440	165	25	0.061
Undarmaa	2010	Japan	Asian	asthma	322	336	243	70	9	242	85	9	0.642
Murk	2011	U.S.A.	mixed	asthma	100	486	91	9	0	452	33	1	0.470
Dmitrieva	2012	Russia	Caucasian	asthma	224	172	217	7	0	170	2	0	1.000
Ungvari	2012	Hungary	Caucasian	asthma	436	765	418	17	1	723	38	4	0.004
Zheng	2012	China	Asian	asthma	198	110	126	61	11	76	29	5	0.325
Chen	2014	China	Asian	asthma	46	52	38	6	2	38	6	8	<0.001
Wan	2014	China	Asian	asthma	58	50	41	16	1	47	3	0	1.000
Ramphul	2014	India	Asian	asthma	192	188	170	21	1	163	24	1	0.605
Amo	2016	Spain	Caucasian	rhinitis	366	526	330	36	0	487	39	0	1.000
Amo	2016	Spain	Caucasian	asthma rhinitis	149	526	146	3	0	487	39	0	1.000
Hua	2016	China	Asian	asthma	1000	1000	659	276	65	688	289	23	0.252
Yang	2017	China	Asian	asthma	74	110	38	31	5	77	30	3	1.000

Author	Year	Country	Ethnicity	Atopy	Sample size (*n*)		Genotype frequency (*n*)						HWE (*P*)
					case	control	Case			control			
							CC	CT	TT	CC	CT	TT	
*FcεRIβ* gene C-109T polymorphism													
Dickson	1999	Australia	Caucasian	asthma	44	26	11	17	16	6	15	5	0.428
Cui	2003	China	Asian	asthma	216	198	23	106	87	19	103	76	0.059
Gan	2004	China	Asian	asthma	45	45	10	12	23	12	14	19	0.015
Zhao	2004	China	Asian	asthma	126	87	11	69	46	9	38	40	0.996
Hizawa	2006	Japan	Asian	asthma	374	374	39	178	157	49	169	156	0.762
Kim	2006	Korea	Asian	asthma	302	264	17	139	146	23	128	113	0.114
Potaczek	2007	Poland	Caucasian	asthma	154	154	25	72	57	27	70	57	0.495
Kim	2009	Korea	Asian	asthma	346	303	20	167	159	28	135	140	0.576
Sharma	2009	India	Asian	asthma	237	221	89	108	40	34	118	69	0.156
Tikhonova	2010	Russia	Caucasian	asthma	140	136	18	69	53	18	70	48	0.339
Ramphul	2014	India	Asian	asthma	189	188	55	99	35	66	87	35	0.505
Wan	2014	China	Asian	asthma	58	50	2	25	31	1	16	33	1.000
Amo	2016	Spain	Caucasian	asthma rhinitis	366	526	78	188	100	105	277	144	0.176
Amo	2016	Spain	Caucasian	rhinitis	149	526	35	67	47	105	277	144	0.176
Hua	2016	China	Asian	asthma	1000	1000	148	436	416	124	470	406	0.502

Abbreviation: HWE, Hardy–Weinberg equilibrium.

First, several relevant studies that met the inclusion criteria were missed in Guo *et al*.’s paper [2–15]. Of the 14 missed studies, 5 articles were published before January 2000 [2–6], which was the start time of published paper restricted in Guo *et al*.’s literature searching strategy [1]; 3 reports were from Japan [2,6,11], 4 studies were from China [9,13–15], 1 each was from South Africa [3], Switzerland [4], Australia [5], India [7], South Korea [8], the U.S.A. [11], and Hungary [12], respectively. In Green *et al.*’s study, black and white populations were recruited, respectively [3]. In Undarmaa *et al.*’s report, children and adult populations were collected, respectively [10].

Second, several studies published by the same research group were included in Guo *et al*.’s report [1]. According to the inclusion and exclusion criteria, when more than two studies were reported by the same research group, only the paper with the largest sample size was included in the analysis. We think Cui *et al.*’s study [16], published in 2004, with 106 adult asthmatics and 106 controls, were incorporated into their another paper, published in 2003, with 216 (number including adults and children) cases and 198 controls [17]. Similarly, the study populations in Hua *et al.*’s papers [18,19] and the Chinese Han case/control populations in Ramphul *et al.*’s article [20], were recruited by the same research group, the two smaller sample-size studies should be excluded from the analysis [18,20].

Third, one study reported by Laprise *et al*. [21], with atopic/non-atopic contrast groups, not all the subjects in atopic group met with the diagnosis criteria of asthma, should be excluded from the analysis.

Fourth, the reported genotype frequency for the C-109T or E+237G polymorphisms of *FcεRIβ* gene in two studies of Guo *et al*.’s paper [1] were not in agreement with the ones in their original papers [22,23]. In Sharma and Ghosh’s study, the CC, CT, and TT genotype frequency for C-109T polymorphism in case/control groups were (89, 108, and 40)/(34, 118, and 69), respectively [22], which were wrongly counted as (87, 113, and 37)/(39, 108, and 74), respectively, in Guo *et al*.’s paper [1]. In Amo *et al*.’s published article, the EE, EG, and GG genotype frequency in control group for E+237G polymorphism were 487, 39, and 0, respectively [23], which were wrongly counted as 144, 277, and 105, respectively [1].

Considering the above-listed mistakes or errors in Guo *et al.*’s published paper, it seems that the findings and conclusions of Guo *et al.*’s study were not entirely reliable [1]. To overcome the limitations, we performed an updated meta-analysis to re-assess the associations of C-109T and E+237G polymorphisms in the *FcεRIβ* gene with allergic disease (asthma and allergic rhinitis) risk. The statistical analysis methods and software used in this comment were the same as reported by Guo *et al*., unless otherwise indicated [1].

The main characteristics of the eligible studies [2–17,19,20,22–42], including the first author, publication year, country where individual study was conducted, ethnicity of study population, atopic disease category, sample size of case/control groups, the detailed genotype frequency, and the *P*-values for Hardy–Weinberg Equilibrium (HWE) test, were shown in Table 1. There were 36 case–control studies about the association between E+237G variant and allergic diseases risk [2–4,6–15,17,19,20,23–28,30–33,36,38,39,41,42], and 15 were about the correlation of C-109T polymorphism with allergic diseases risk [5,8,12,14,17,19,22,23,29,34,35,37,38,40]. Of the 15 case–control studies about C-109T polymorphism and allergic disease risk (14 ones according to ethnicity or HWE classification), 10 were performed in Asians [8,14,17,19,20,22,29,34,35,38] and 4 were conducted in Caucasians [5,23,37,40], respectively; 13 studies were about asthma risk [5,8,17,19,20,22,29,34,37,38,40], 1 was about allergic rhinitis risk [23], and 1 about asthma and rhinitis risk [23], respectively; genotype frequency distribution in control groups of 13 studies were in agreement with HWE [5,8,14,17,19,20,22,23,34,35,37,38,40] and 1 was not [29], respectively. Of the 36 case–control studies about E+237G variant with allergic diseases risk (35 ones according to ethnicity or HWE classification), 25 were carried out in Asians [2,6–10,13–15,17,19,20,24,26–29,32,33,36,38,39,42], 8 were performed in Caucasians [3,4,12,23,25,30,31,41], 1 in Africans [3] and 1 in mixed populations [11], respectively; 31 studies were about asthma risk [2–4,6–15,17,19,20,24–26,28,29,31–33,36,38,39,41,42], 2 were on rhinitis risk [23,27], and 3 were concerned with asthma/rhinitis risk [23,30,31], respectively; genotype frequency distribution in control groups of 32 studies were in line with HWE [2–4,6–11,13–15,17,19,20,23–26,28–33,36,38,39,41,42] and 3 were not [12,13,27], respectively.

Table 2 listed the summary odds ratios (ORs) of the association of *FcεRIβ* C-109T polymorphism with allergic diseases risk. Overall, no significant associations between C-109T polymorphism and allergic diseases risk were observed (OR = 1.001, 95% confidence interval (CI): 0.909–1.102 for CC+CT vs. TT and OR = 1.015, 95% CI: 0.788–1.307 for CC vs. CT+TT, respectively). When subgroup analyses by ethnicity (Asian and Caucasian), allergic disease classification (asthma, rhinitis, and both) and HWE (in and not) were performed, we did not find any statistically significant associations of C-108T polymorphism with allergic diseases risk (Table 2). No any publication and other small study related biases were observed in overall and subgroup analyses (Table 2).

**Table 2:** Summary ORs for the association between *FcεRIβ* C-109T polymorphism and allergic diseases risk

Comparisons	Sample size	Number of studies	Hypothesis tests			Heterogeneity tests			Publication bias test (*P*)	
	Case/control		OR (95% CI)	*z*	*P*	χ^2^ (df)	*P*	*I^2^* (%)	Begg’s test	Egger’s test
Overall										
C vs. T	7492/7144	14	1.024 (0.900–1.164)	0.36	0.722	37.83 (13)	<0.001	65.6	0.784	0.958
CC vs. TT	1994/1862	14	1.007 (0.759–1.335)	0.05	0.963	36.77 (13)	<0.001	64.6	0.870	0.582
CC vs. CT	2333/2231	14	1.028 (0.807–1.311)	0.22	0.823	30.59 (13)	0.004	57.5	0.702	0.419
CT vs. TT	3165/3051	14	0.984 (0.890–1.089)	0.31	0.758	14.33 (13)	0.351	9.3	0.547	0.538
CC+CT vs. TT	3746/3572	14	1.001 (0.909–1.102)	0.01	0.989	21.72 (13)	0.060	40.1	0.784	0.670
CC vs. CT+TT	3746/3572	14	1.015 (0.788–1.307)	0.11	0.911	37.20 (13)	<0.001	65.1	0.956	0.446
Stratification by ethnicity										
Asians										
C vs. T	5786/5460	10	1.052 (0.883–1.254)	0.57	0.567	36.51 (9)	<0.001	75.3	0.655	0.802
CC+CT vs. TT	2893/2730	10	1.070 (0.895–1.280)	0.74	0.458	18.97 (9)	0.025	52.6	0.325	0.304
CC vs. CT+TT	2893/2730	10	0.998 (0.695–1.434)	0.01	0.992	36.70 (9)	<0.001	75.5	0.788	0.537
Caucasians										
C vs. T	1706/1684	4	0.984 (0.858–1.127)	0.24	0.813	0.89 (3)	0.828	<0.1	0.042	0.036
CC+CT vs. TT	853/842	4	0.919 (0.747–1.130)	0.80	0.422	1.99 (3)	0.576	<0.1	0.174	0.201
CC vs. CT+TT	853/842	4	1.067 (0.836–1.362)	0.52	0.601	0.48 (3)	0.924	<0.1	1.000	0.412
Stratification by atopic disease categories										
Asthma										
C vs. T	6462/6092	13	1.024 (0.885–1.185)	0.32	0.750	37.83 (12)	<0.001	68.3	0.903	0.950
CC+CT vs. TT	3231/3046	13	1.032 (0.883–1.207)	0.40	0.691	21.52 (12)	0.043	44.2	1.000	0.712
CC vs. CT+TT	3231/3046	13	0.997 (0.744–1.336)	0.02	0.983	37.13 (12)	<0.001	67.7	0.542	0.472
Stratification by HWE										
C vs. T	7402/7054	13	1.035 (0.907–1.180)	0.51	0.613	36.83 (12)	<0.001	67.4	1.000	0.861
CC+CT vs. TT	3701/3527	13	1.006 (0.913–1.108)	0.11	0.911	21.00 (12)	0.050	42.9	0.272	0.483
CC vs. CT+TT	3701/3527	13	1.026 (0.789–1.335)	0.19	0.848	36.76 (12)	<0.001	67.4	0.807	0.516

Abbreviation: df, degree of freedom.

Table 3 showed the summary ORs for the association between *FcεRIβ* E237G variant and allergic diseases risk. Overall, we observed *FcεRIβ* 237G allele was associated with increased risk of allergic diseases in total population (OR = 1.178, 95% CI: 1.022–1.357 for G vs. E and OR = 1.207, 95% CI: 1.031–1.411 for GG+EG vs. EE, respectively) (Table 3 and Figure 1). When restricted the analysis to the studies with control groups’ genotype frequency distribution were met with HWE, we observed an elevated risk of allergic diseases among subjects carrying EG or GG genotypes, in comparison with EE genotype carriers (OR = 1.225, 95% CI: 1.041–1.442) (Table 3 and Figure 1). When stratified analyses were conducted by ethnicity, we found an increased risk of allergic diseases in subjects carrying EG or GG genotypes, compared with EE genotype carries in Asians (OR = 1.189, 95% CI: 1.001–1.412) (Table 3 and Figure 2). No significant association of E237G polymorphism with allergic diseases risk was observed in Caucasians (OR = 1.544, 95% CI: 0.884–2.697 for G allele vs. E allele and OR = 1.547, 95% CI: 0.895–2.673 for EG+GG vs. EE, respectively) (Table 3 and Figure 2). In subgroup analyses by allergic diseases classification (asthma, allergic rhinitis, and both), we did not observe significant association of E237G with any allergic diseases categories (Table 3 and Figure 3).

**Figure 1:**
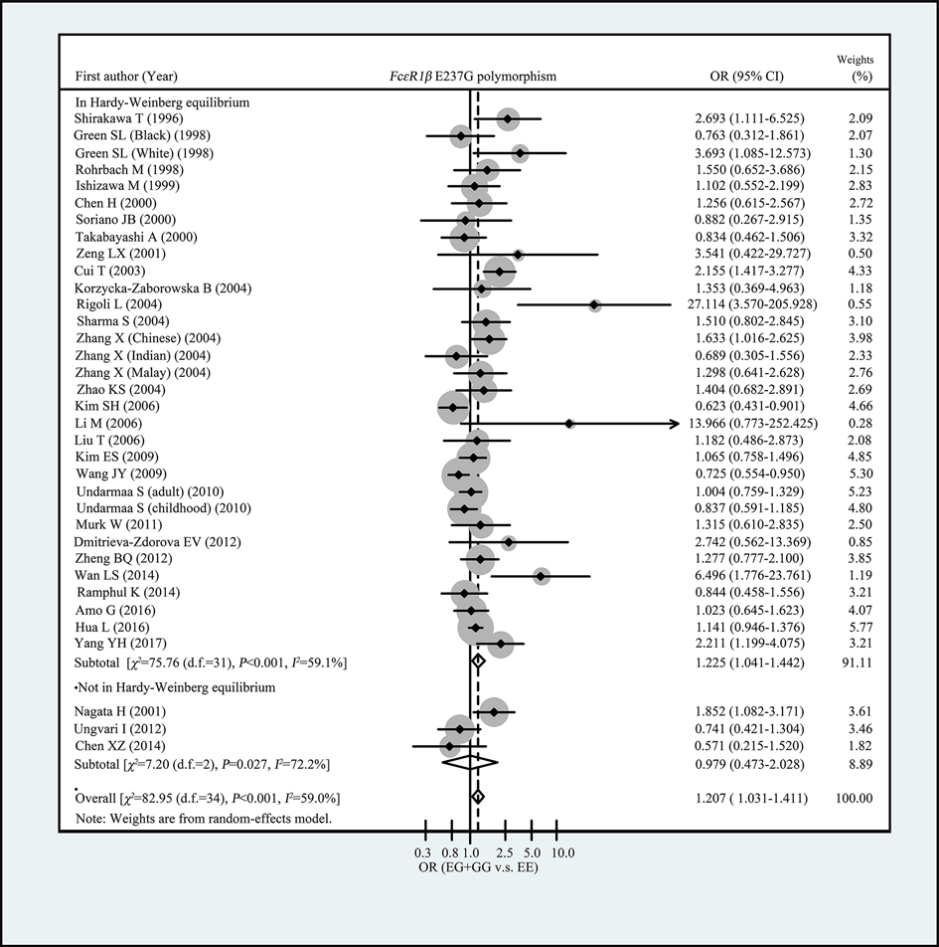
Forest plots for the association of FcεRIβ E237G polymorphism with allergic diseases risk (subgroup analysis by HWE)

**Figure 2:**
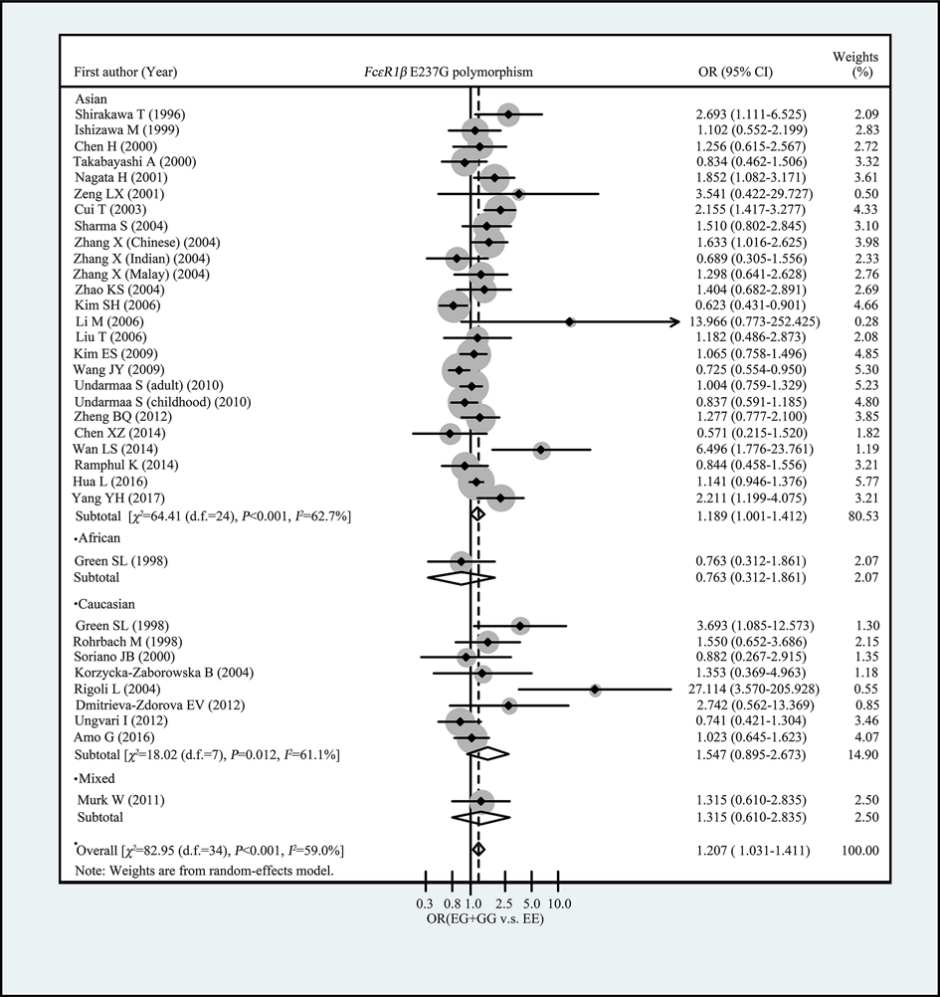
Forest plot for the association of FcεRIβ E237G polymorphism with allergic diseases risk (subgroup analysis by ethnicity)

**Figure 3:**
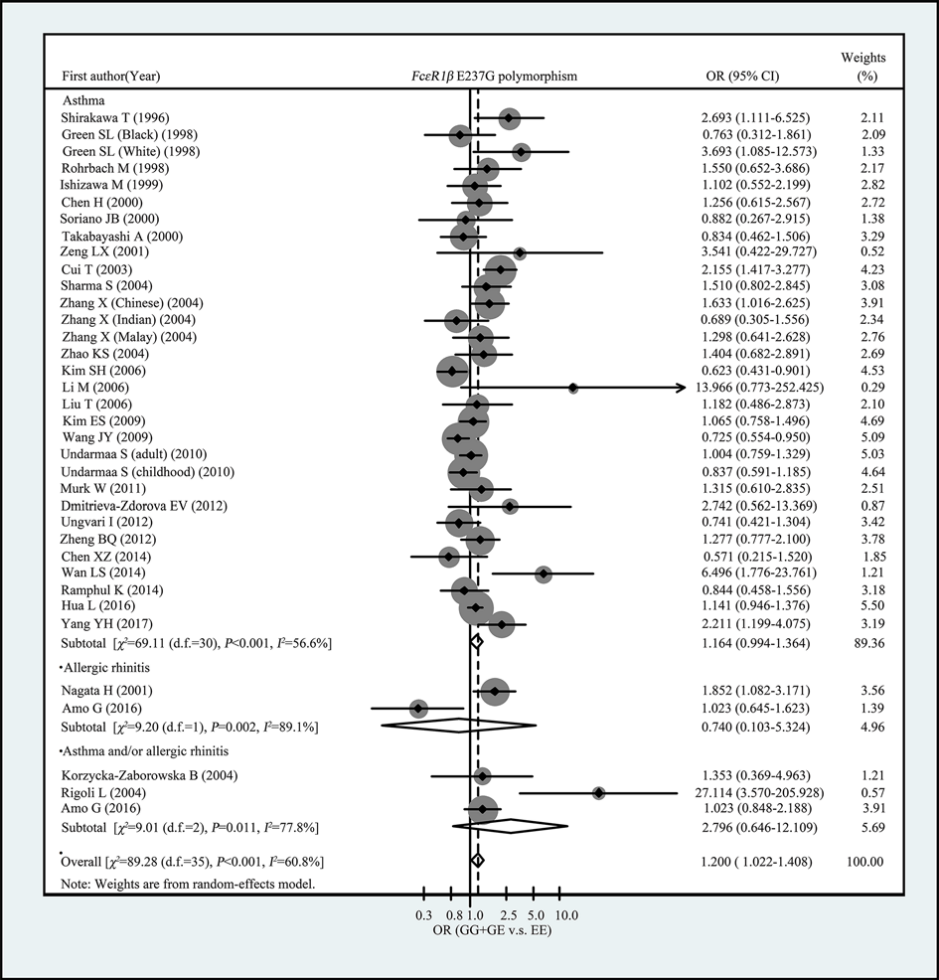
Forest plot for the association of FcεRIβ E237G polymorphism with allergic diseases risk (subgroup analysis by allergy category)

**Table 3:** Summary ORs for the association between *FcεRIβ* E273G polymorphism and allergic diseases risk

Comparisons	Sample size	Number of studies	Hypothesis tests			Heterogeneity tests			Publication bias test (*P*)	
	Case/control		OR (95% CI)	*Z*	*P*	χ^2^ (df)	*P*	*I^2^* (%)	Begg’s test	Egger’s test
Overall										
G vs. E	14552/14956	35	1.178 (1.022–1.357)	2.25	0.024	84.83 (34)	<0.001	59.9	0.028	0.025
GG+GE vs. EE	7276/7478	35	1.207 (1.031–1.411)	2.35	0.019	82.95 (34)	<0.001	59.0	0.024	0.008
Stratification by ethnicity										
Asians										
G vs. E	10694/10080	25	1.158 (0.994–1.350)	1.88	0.060	65.83 (24)	<0.001	63.5	0.176	0.122
GG+GE vs. EE	5347/5040	25	1.189 (1.001–1.412)	1.98	0.048	64.41 (24)	<0.001	62.7	0.148	0.046
Caucasians										
G vs. E	3576/3820	8	1.544 (0.884–2.697)	1.53	0.126	19.63 (7)	0.006	64.3	0.026	0.028
GG+GE vs. EE	1788/1910	8	1.547 (0.895–2.673)	1.56	0.118	18.02 (7)	0.012	61.1	0.026	0.028
Stratification by atopic disease categories										
Asthma										
G vs. E	12660/13324	31	1.148 (0.994–1.326)	1.88	0.060	72.22 (30)	<0.001	58.5	0.051	0.081
GG+GE vs. EE	6330/6662	31	1.164 (0.994–1.364)	1.89	0.059	69.11 (30)	<0.001	56.6	0.047	0.031
Allergic rhinitis										
G vs. E	764/1252	2	0.680 (0.124–3.737)	0.44	0.657	7.30 (1)	0.007	86.3	0.317	-
GG+GE vs. EE	382/626	2	0.740 (0.103–5.324)	0.30	0.765	9.20 (1)	0.002	89.1	0.317	-
Asthma and/or allergic rhinitis										
G vs. E	1128/1432	3	2.955 (0.616–14.181)	1.35	0.176	10.60 (2)	0.005	81.1	0.117	0.449
GG+GE vs. EE	564/716	3	2.796 (0.646–12.109)	1.37	0.169	9.01 (2)	0.011	77.8	0.117	0.451
Stratification by HWE										
Yes										
G vs. E	13122/13122	32	1.211 (1.046–1.403)	2.55	0.011	76.29 (31)	<0.001	59.4	0.009	0.008
GG+GE vs. EE	6561/6561	32	1.225 (1.041–1.442)	2.44	0.015	75.76 (31)	<0.001	59.1	0.011	0.004

Abbreviation: df, degree of freedom.

We also performed a cumulative meta-analysis which accumulated the evidence about association of E237G variant with allergic diseases risk in the order of publication year of individual study. We observed that the association of EG/GG genotypes with increased allergic diseases started to become significant for the first time when Zeng *et al.*’s [28] study published in the year of 2001 (OR = 1.374, 95% CI: 1.013–1.864) and the summary OR became very similar to the OR estimated in this report (OR = 1.207) when Wang *et al.*’s [39] study published in the year of 2009 (OR = 1.299, 95% CI: 1.026–1.644) (Figure 4).The overall tendency of summary OR variation seemed alarming at the top of forest plot of the cumulative meta-analysis. It should be noted that Shirakawa *et al*. [2] (study 1) reported the first positive association of 237EG+GG with asthma risk with OR being equal to 2.693 in a Japanese (Asian) population and the second included study [3] with two independent case–control studies [one in black (study 2) and one in white (study 3)] reported different associations, one is negative association of 237EG+GG with asthma risk in Black (OR = 0.763) and the other is similar to Shirakawa *et al*.’s result in White population (OR = 3.693). When merging the result of study 1 with study 2, the overall OR significantly decreased. And when summarizing the results of the first three studies (*i.e.* study 1, 2, and 3) the overall OR looked bigger than the second. As a result, the overall ORs would generate a V-form change in the forest plot of cumulative meta-analysis. The alarming changes in the plot of cumulative meta-analysis also indicated that there was high heterogeneity of the results of the included individual studies.

**Figure 4:**
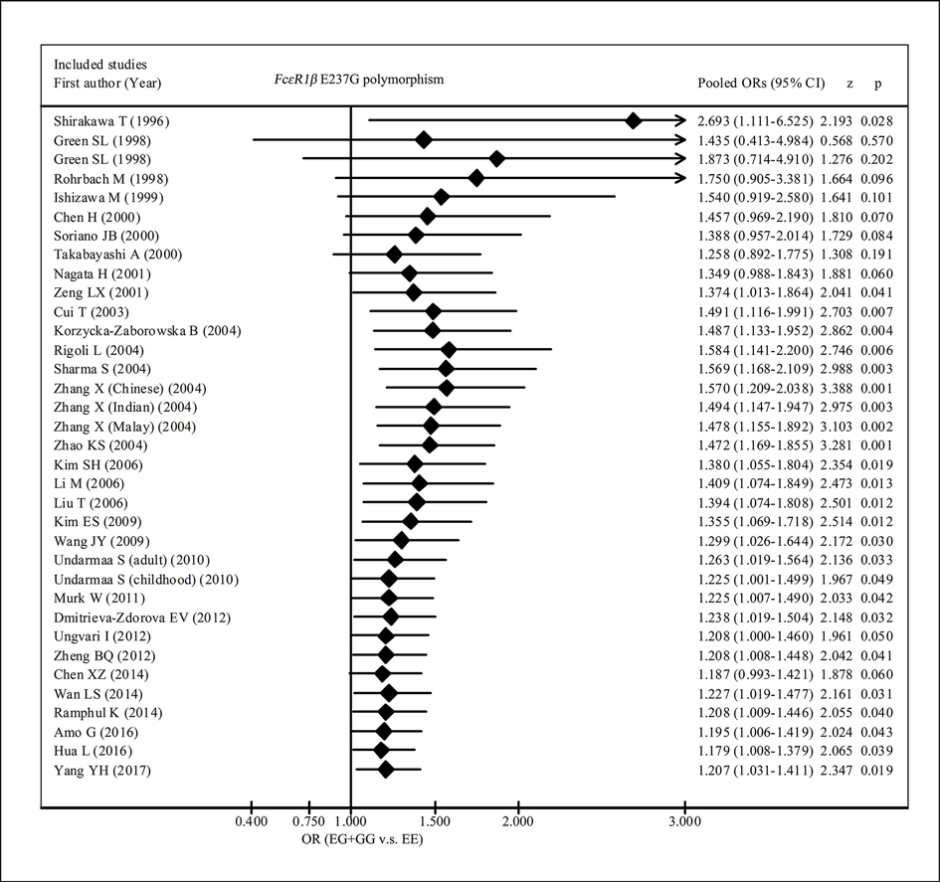
Forest plot of cumulative meta-analysis for the association of FcεRIβ E237G polymorphism with allergic diseases risk

Sensitivity analysis was performed by sequentially omitting each individual study in the order of publication year and the pooled ORs were estimated repeatedly, which was used to evaluate the stability of the results of present meta-analysis. The sensitivity analysis showed that the association of EG and GG genotypes with increased risk of allergic diseases maintained statistically significant when removing any each individual study (Figure 5). Egger’s regression test and Begg’s rank correlation test were used to evaluate the small-study effects and potential publication bias in current meta-analysis. Both tests indicated that the significant association of G allele or EG+GG genotypes with elevated risk of allergic diseases might strongly influenced by small-study effect or publication bias (Table 3). The Egger’s funnel plots for the association between E237G polymorphism and allergic diseases risk also showed that the OR distributions for both G allele vs. E allele (Figure 6A) and EG+GG vs.EE (Figure 6B) were obviously asymmetrical.

**Figure 5:**
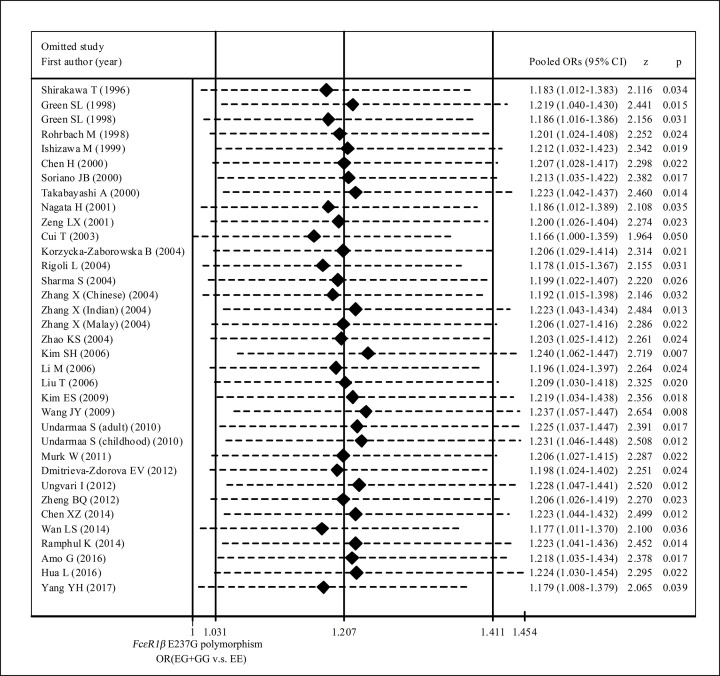
Sensitivity analysis for the association between FcεRIβ E237G polymorphism and allergic diseases risk

**Figure 6:**
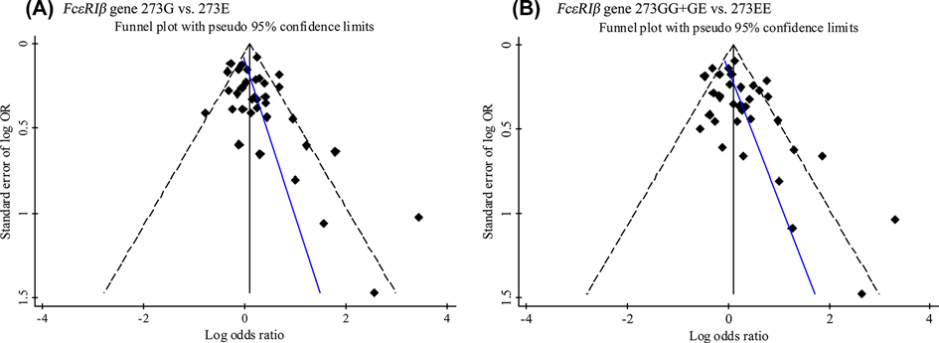
Egger’s funnel plots for the association between FcεRIβ E237G polymorphism and allergic diseases risk (**A**) G allele vs. E allele; (**B**) EG/GG genotypes vs. EE genotype.

There are some inherent limitations of meta-analysis which should be taken into consideration when using the results of this comment. First, there was high heterogeneity in this meta-analysis, especially in the case of association of E237G variant with allergic diseases risk. Although, subgroup analyses were performed on the basis of ethnicity, allergic disease category and HWE, heterogeneity among the included studies still be statistically significant in all subgroups. Second, publication bias tests indicated that the probable existence of publication bias, i.e*.* some unpublished negative results studies thus could not be included in this analyses might result in an over-estimated association of E237G with allergic disease risk.

In conclusion, the results of Guo et al*.*’s study [1] should be interpreted with caution. To make an asserted conclusion, well-designed studies with large number of homogeneous population are required. We do hope that this comment will be helpful to clarify the results presented by Guo et al. [1].

## Abbreviations

CI: confidence interval
FcεRIβ: high-affinity IgE receptor β chain
HWE: Hardy–Weinberg equilibrium
OR: odds ratio
